# Supplementary material for: The role of auxiliary domains in modulating CHD4 activity suggests mechanistic commonality between enzyme families
Source: Nat Commun. 2022 Dec 6;13:7524. doi: 10.1038/s41467-022-35002-0 (PMC9726900; doi:10.1038/s41467-022-35002-0)
Supplement: Supplementary file 1 — Supplrmentary Information [file 41467_2022_35002_MOESM1_ESM.pdf]

Supplementary Figures

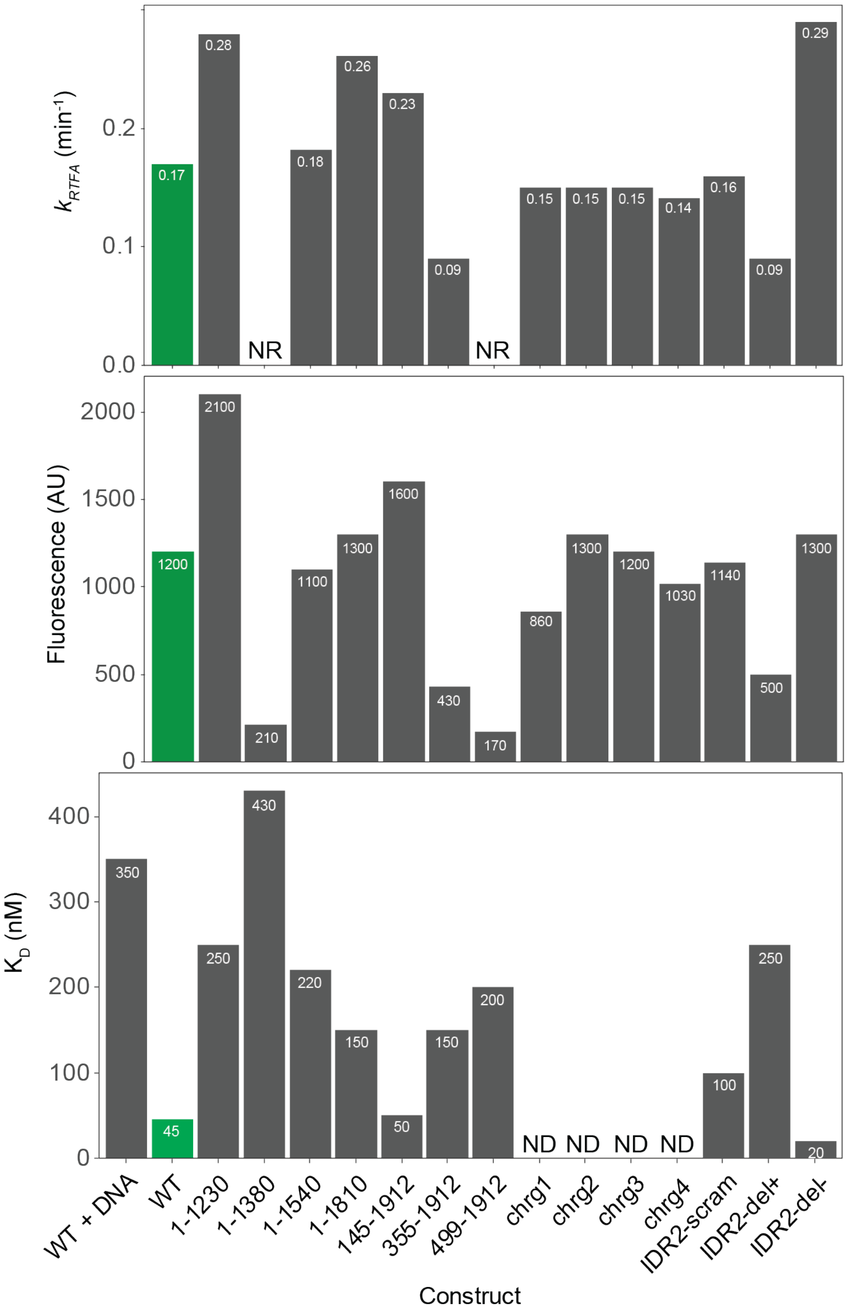

Supplementary Figure 1. Summary of RTFA and MST data for all CHD4 variants used in this study. Pseudo-first order rate constants and final fluorescence values were obtained by fitting the data to the asymptotic exponential equation $y = F_{lim}(1 - \exp(-k_{RTFA} * t))$, where F_{lim} is the limiting value of the fluorescence, k_{RTFA} is a pseudo-first-order rate constant (units of min^{-1}), and t is the time in minutes after addition of CHD4 and ATP. NR indicates data that could not be reliably fitted. ND indicates that no data were recorded in these instances. All experiments were conducted once with at least 3 technical replicates, and fitting was performed upon the averages of replicates. We estimate the uncertainty in these parameters to be ~25%.

A

```
              30         40         50         60         70         80         90        100
  WT  DALLNNSLPP PHPENEEDPE EDLSETETPK LKKKKKPKKP RDPKIPKSKR QKKERMLLCR QLGDSSGEGP EFVEEEEEVA
chrg  DALLNNSLPP PHPENEEDPE EDLSETETPA TSASTTASTP RDPKIPKSKR QKKERMLLCR QLGDSSGEGP EFVEEEEEVA
                                         chrg1

             110        120        130        140        150        160
  WT  LRSDSEGSDY TPGKKKKKKL GPKKEKKSKS KRKEEEEEED DDDDSKEPKS SAQLLEDWGM
chrg  LRSDSEGSDY TPGASTASTA GPKKEKKSKS KRKSTTASSA TSTASKEPKS SAQLLEDWGM
                    chrg2                  chrg3
```

B

```
             236        246        256        266        276        286        296        306
   WT  AAAAAAAVAV VESMVTATEV APPPPPVEVP IRKAKTKEGK GPNARRKPKG SPRVPDAKKP KPKKVAPLKI KLGGFGSKRK
scram  EEKPPEPDAA IVSSKASGNR IRKPGEPPVK SYDMGRAAVA SDVKAKTDEV KLPVFASVVT KLGKASPSGD KPRRKANKAS
 del+  MDVFMKGLSK AKEGVVAAAE KTKQGVAEAA GKTKEGVLYV GSKTKEGVVH GVATVAEKTK EQVTNVGGAV VTGVTAVAQK
 del-  MSVFMKGLSK AKKGVVAAAH KTKQGVARAA GKTKRGVLYV GSKTASGVVH GVATVAHKTK KQVTNVGGAV VTGVTAVAQK

             316        326        336        346        356
   WT  RSSSEDDDLD VESDFDDASI NSYSVSDGST SRSSRSRKKL RTTKKKKKGE EEVTAV
scram  TLRDIKKTKV KDSAPPPKKA GEVKDKSFAK RPTEGERGRS LVRSASKEVS VATSDT
 del+  TVEGAGSIAA ATGFVKKDQL GKNEEGAPQE GILEDMPVDP DNEAYEMPSE EGYQDY
 del-  TVRGAGSIAA ATGFVKKKQL GKNHRGAPQK GILKRMPVDP DNSAYKMPSH RGYQHY
```

Supplementary Figure 2. Replacement sequences for IDR1 and IDR2 mutants. **A**. Amino acid sequences of the WT IDR1 (residue 21–160) and chrg mutants in which the charged patches (chrg1–3) were replaced with A, T or S. **B**. Amino acid sequences of wildtype IDR2 (residues 227–362) and for the sequences used in replacement experiments.

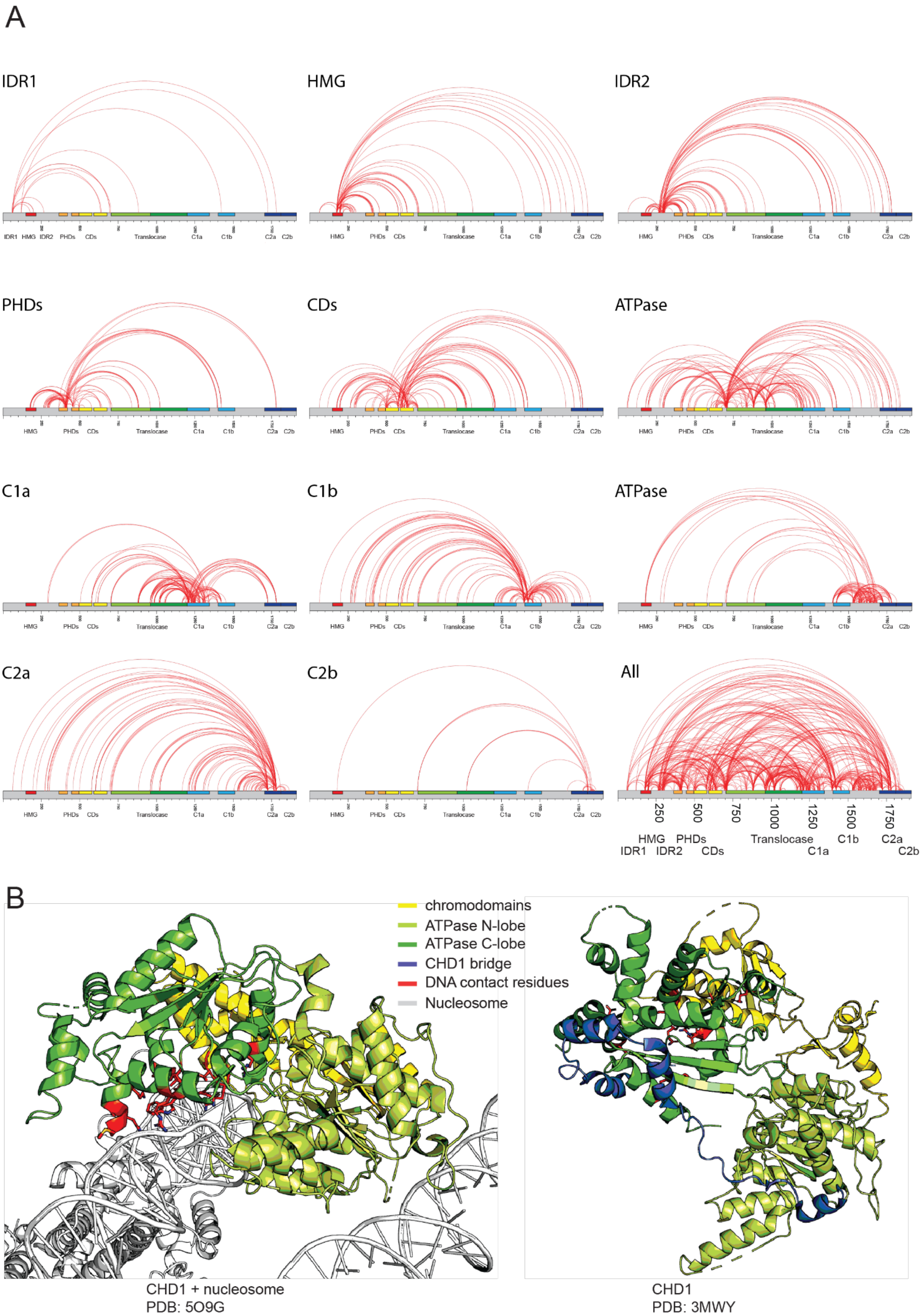
A
IDR1
HMG
IDR2
PHDs
CDs
ATPase
C1a
C1b
ATPase
C2a
C2b
All
250
500
750
1000
1250
1500
1750
HMG PHDs Translocase C1b C2a
IDR1 IDR2 CDs C1a C2b
B
chromodomains
ATPase N-lobe
ATPase C-lobe
CHD1 bridge
DNA contact residues
Nucleosome
CHD1 + nucleosome
PDB: 5O9G
CHD1
PDB: 3MWY

Supplementary Figure 3. XLMS data. A. Covalent crosslinks observed for CHD4 divided into subsets by region; the final image shows all observed XLs. **B.** *Left*: Portion of the cryoEM structure of CHD1 bound to a nucleosome (PDB: 5O9G, {Farnung, 2017 #811}). The C-terminal bridge is not resolved in this structure. *Right*: X-ray crystal structure of the ATPase domain of CHD1 (PDB: 3MWY, {Hauk, 2010 #19}). The C-lobe is shown in the same orientation as in 5O9G and the 'opening up' of the N-lobe is apparent. The construct included the equivalent of the CHD4 C1a region, which is referred to in CHD1 as the C-terminal bridge (*yellow*). This region contacts both lobes of the DNA translocase domain.

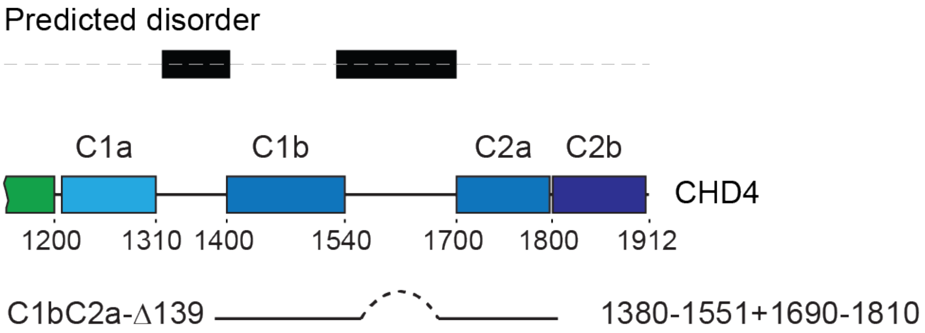

Supplementary Figure 4. Schematic of the Ca1C2a construct used for crystallography of the C1b-C2a region.

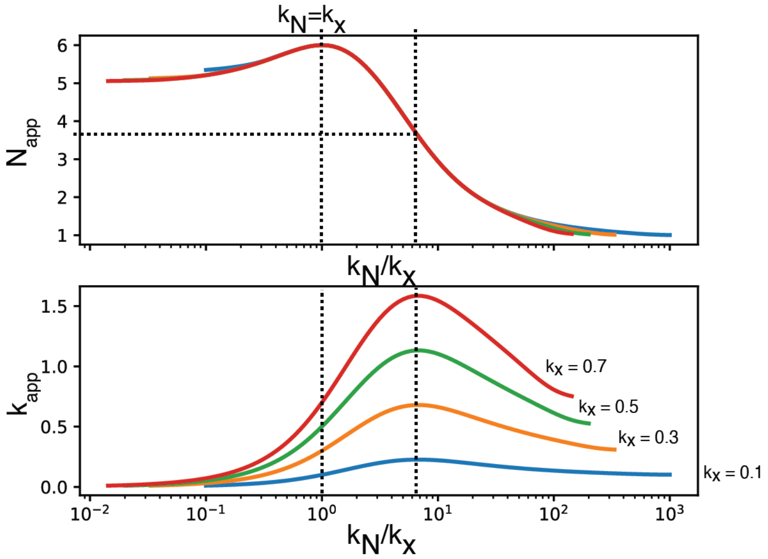

Supplementary Figure 5. The effect of an additional reaction step on the observed kinetic behaviour for remodelling. Numerical simulations show the effect of an additional reaction step (with rate constant k_X – on top of the multiple identical steps that occur with a rate constant of k_N) on the observed kinetic parameters for remodelling. Simulations were carried out for N = 5, across multiple values of k_X. As the relative values of the two rate constants change – because of changes in ATP concentration or in the CHD4 construct used – the apparent rate constant and *N* value both change significantly. When the rate of the multiple identical steps (k_N) is low and rate limiting (*left hand side* of graphs), N_{app} tends to a value equal to the number of identical steps (*top graph*) and k_{app} tends to k_N. When $k_N >> k_X$, k_X becomes rate limiting so that N_{app} tends to 1. In the central region, when k_N and k_X are similar, N_{app} tends to a value one larger than the number of identical reaction steps.

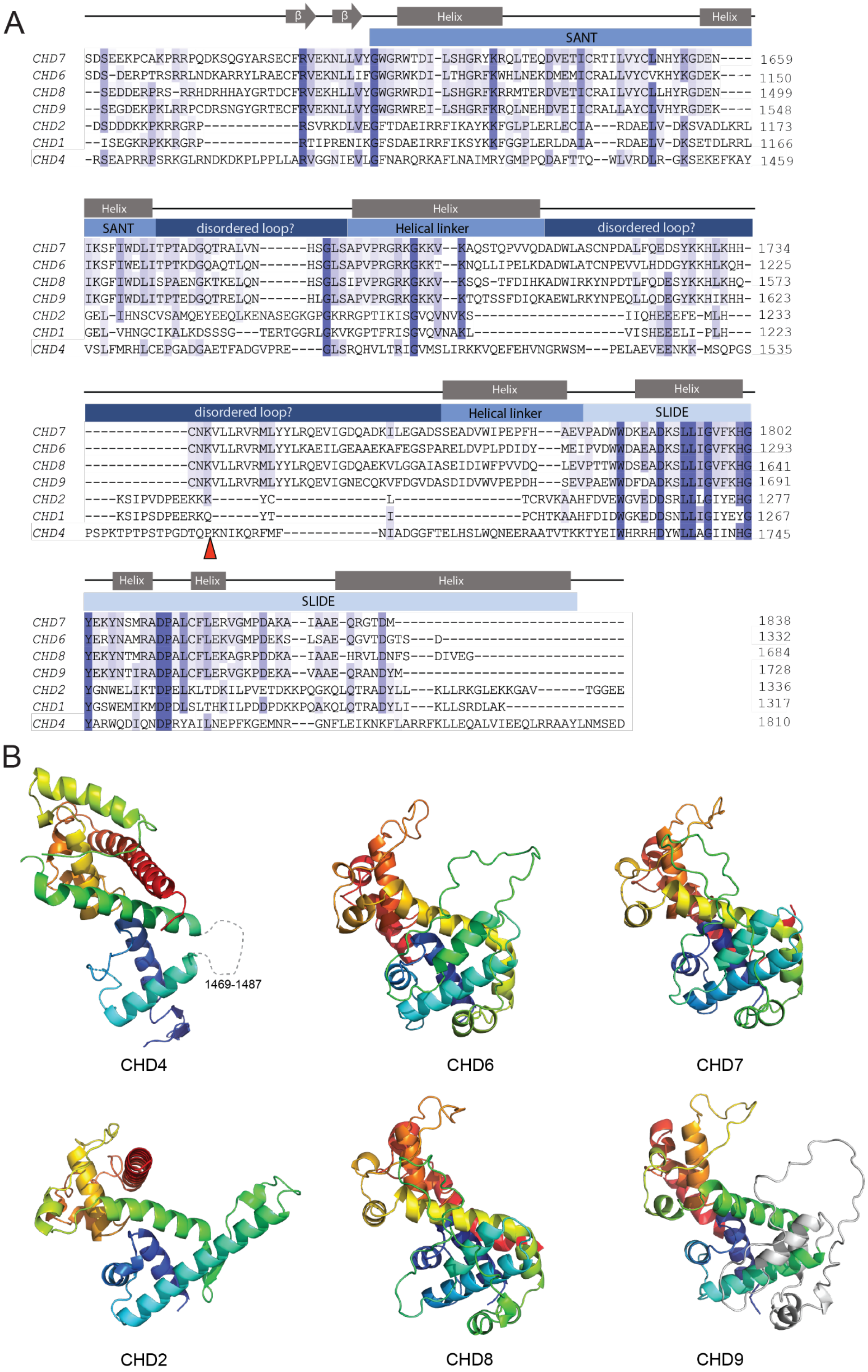
A
β
β
Helix
Helix
SANT
CHD7 SDSEEKPCAKPRRPQDKSQGYARSECFRVEKNLLVYGWGRWTDI-LSHGRYKRQLTEQDVETICRTILVYCLNHYKGDEN---- 1659
CHD6 SDS-DERPTRSRRLNDKARRYLRAECFRVEKNLLIFGWGRWKDI-LTHGRFKWHLNEKDMEMICRALLVYCVKHYKGDEK---- 1150
CHD8 --SEDDERPRS-RRHDRHHAYGRTDCFRVEKHLLVYGWGRWRDI-LSHGRFKRRMTERDVETICRAILVYCLLHYRGDEN---- 1499
CHD9 --SEGDEKPKLRRPCDRSNGYGRTECFRVEKNLLVYGWGRWREI-LSHGRFKRQLNEHDVEIICRALLAYCLVHYRGDEK---- 1548
CHD2 -DSDDDKKPKRRGRP------------RSVRKDLVEGFTDAEIRRFIKAYKKFGLPLERLECIA---RDAELV-DKSVADLKRL 1173
CHD1 --ISEGKRPKKRGRP------------RTIPRENIKGFSDAEIRRFIKSYKKFGGPLERLDAIA---RDAELV-DKSETDLRRL 1166
CHD4 -RSEAPRRPSRKGLRNDKDKPLPPLLARVGGNIEVLGFNARQRKAFLNAIMRYGMPPQDAFTTQ--WLVRDLR-GKSEKEFKAY 1459
Helix
Helix
SANT
disordered loop?
Helical linker
disordered loop?
CHD7 IKSFIWDLITPTADGQTRALVN------HSGLSAPVPRGRKGKKV--KAQSTQPVVQDADWLASCNPDALFQEDSYKKHLKHH- 1734
CHD6 IKSFIWELITPTKDGQAQTLQN------HSGLSAPVPRGRKGKKT--KNQLLIPELKDADWLATCNPEVVLHDDGYKKHLKQH- 1225
CHD8 IKGFIWDLISPAENGKTKELQN------HSGLSIPVPRGRKGKKV--KSQS-TFDIHKADWIRKYNPDTLFQDESYKKHLKHQ- 1573
CHD9 IKGFIWDLITPTEDGQTRELQN------HLGLSAPVPRGRKGKKV--KTQTSSFDIQKAEWLRKYNPEQLLQDEGYKKHIKHH- 1623
CHD2 GEL-IHNSCVSAMQEYEEQLKENASEGKGPGKRRGPTIKISGVQVNVKS------------------IIQHEEEFE-MLH--- 1233
CHD1 GEL-VHNGCIKALKDSSSG---TERTGGRLGKVKGPTFRISGVQVNAKL------------------VISHEEELI-PLH--- 1223
CHD4 VSLFMRHLCEPGADGAETFADGVPRE----GLSRQHVLTRIGVMSLIRKKVQEFEHVNGRWSM---PELAEVEENKK-MSQPGS 1535
Helix
Helix
disordered loop?
Helical linker
SLIDE
CHD7 -------------CNKVLLRVRMLYYLRQEVIGDQADKILEGADSSEADVWIPEPFH---AEVPADWWDKEADKSLLIGVFKHG 1802
CHD6 -------------CNKVLLRVRMLYYLKAEILGEAAEKAFEGSPARELDVPLPDIDY---MEIPVDWWDAEADKSLLIGVFKHG 1293
CHD8 -------------CNKVLLRVRMLYYLRQEVIGDQAEKVLGGAIASEIDIWFPVVDQ---LEVPTTWWDSEADKSLLIGVFKHG 1641
CHD9 -------------CNKVLLRVRMLYYLKQEVIGNECQKVFDGVDASDIDVWVPEPDH---SEVPAEWWDFDADKSLLIGVFKHG 1691
CHD2 ----KSIPVDPEEKKK------YC-------------L---------------TCRVKAAHFDVEWGVEDDSRLLLGIYEHG 1277
CHD1 ----KSIPSDPEERKQ------YT-------------I---------------PCHTKAAHFDIDWGKEDDSNLLIGIYEYG 1267
CHD4 PSPKTPTPSTPGDTQPKNIKQRFMF-----------NIADGGFTELHSLWQNEERAATVTKKTYEIWHRRHDYWLLAGIINHG 1745
Helix
Helix
Helix
SLIDE
CHD7 YEKYNSMRADPALCFLERVGMPDAKA--IAAE-QRGTDM--------------------------- 1838
CHD6 YERYNAMRADPALCFLEKVGMPDEKS--LSAE-QGVTDGTS---D---------------------- 1332
CHD8 YEKYNTMRADPALCFLEKAGRPDDKA--IAAE-HRVLDNFS---DIVEG------------------ 1684
CHD9 YEKYNTIRADPALCFLERVGKPDEKA--VAAE-QRANDYM--------------------------- 1728
CHD2 YGNWELIKTDPELKLTDKILPVETDKKPQGKQLQTRADYLL---KLLRKGLEKKGAV------TGGEE 1336
CHD1 YGSWEMIKMDPDLSLTHKILPDDPDKKPQAKQLQTRADYLI---KLLSRDLAK-------------- 1317
CHD4 YARWQDIQNDPRYAILNEPFKGEMNR---GNFLEIKNKFLARRFKLLEQALVIEEQLRRAAYLNMSED 1810
B
1469-1487
CHD4
CHD6
CHD7
CHD2
CHD8
CHD9

Supplementary Figure 6. All CHD family members are predicted to contain a SANT-SLIDE domain. A. Partial sequence alignments of the indicated human CHD-family proteins. The secondary structure of the CHD4 SANT-SLIDE domain is indicated above the alignment. There is higher sequence similarity in the SLIDE region compared to the SANT region. **B.** Comparison of the CHD4 SANT-SLIDE domain with AlphaFold2 predictions of the corresponding regions of the indicated CHD-family proteins. In each case other than CHD9, the chain is coloured from N- to C-terminus using a blue-red rainbow scheme. For CHD6–9, the 1469–1487 loop of CHD4 is replaced with an 80-residue loop. For CHD9, this long loop is coloured *grey,* and the remainder of the chain is coloured in the rainbow format, better highlighting the topological similarity between CHD7–9 and CHD4.

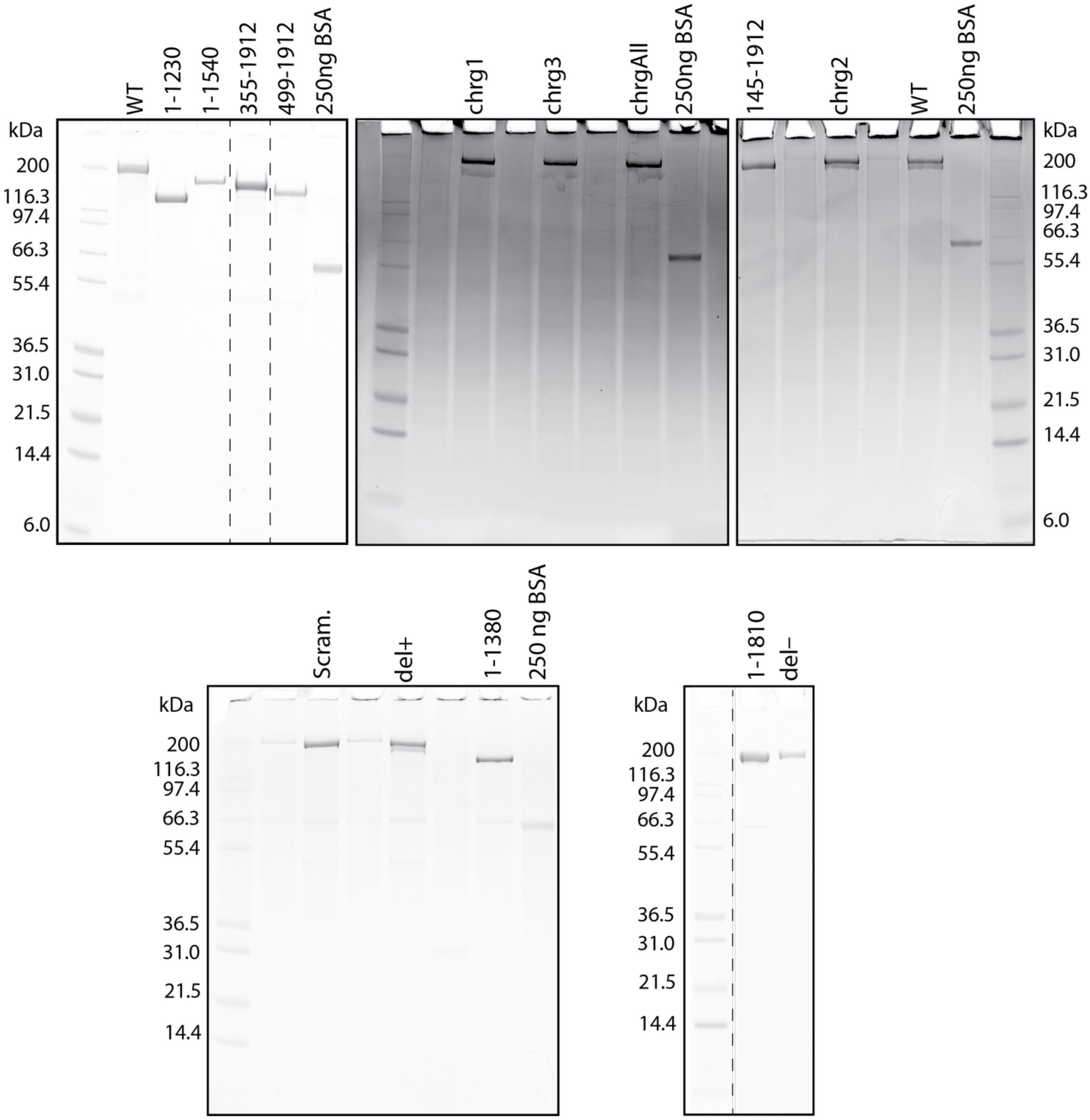

Supplementary Figure 7. Assessment of purity of recombinant CHD4 polypeptides. FLAG-tagged CHD4 constructs were cloned into pcDNA3.1,expressed in HEK293 Expi cells and then purified with one-step FLAG-affinity pulldown. The final products were loaded onto SDS-PAGE gels for quality control; the protein concentrations were quantified with densitometry using 250 ng BSA as references. Representative gels are shown; each protein was purified at least twice.

Supplementary Data 1. CHD4 XLMS data. See separate file.

Supplementary Table 1. Crystal and X-ray diffraction data for CHD4-C1bC2a

	Apo structure
Wavelength	0.9537
Resolution range	48.80 -2.90 (3.07-2.90)
Space group	P 2 1
Unit cell	65.43, 66.67, 99.37, β=100.77
Total reflections	82,638 (12,751)
Unique reflections	18,602 (2,791)
Multiplicity	4.44 (4.56)
Completeness (%)	98.0 (91.7)
Mean I/sigma(I)	9.28 (1.71)
Wilson B-factor	76.2
R-merge	0.116 (81.1)
R-meas	0.131 (91.6)
CC1/2	0.994 (0.706)
Reflections used in refinement	18443 (1708)
Reflections used for R-free	902 (81)
R-work	0.2629 (0.3436)
R-free	0.3039 (0.3490)
Number of non-hydrogen atoms	3557
macromolecules	3551
ligands	0
solvent	6
Protein residues	427
RMS (bonds)	0.008
RMS (angles)	1.15
Ramachandran favored, allowed, outliers (%)	96.11, 3.89, 0.00
Rotamer outliers (%)	0.27
Average B-factor	90.07
macromolecules	90.12
ligands	0
solvent	65.96

Statistics for the highest-resolution shell are shown in parentheses

References

1. Zhong, Y., et al., *CHD4 slides nucleosomes by decoupling entry- and exit-side DNA translocation.* Nat Commun, 2020. **11**(1): p. 1519.
2. Hagman, J.R., et al., *Chromodomain helicase DNA-binding 4 (CHD4) regulates early B cell identity and V(D)J recombination.* Immunol Rev, 2022. **305**(1): p. 29-42.
3. Weiss, K., et al., *Correction: The CHD4-related syndrome: a comprehensive investigation of the clinical spectrum, genotype-phenotype correlations, and molecular basis.* Genet Med, 2020. **22**(3): p. 669.
4. Hauk, G., et al., *The chromodomains of the Chd1 chromatin remodeler regulate DNA access to the ATPase motor.* Mol Cell, 2010. **39**(5): p. 711-23.
5. Farnung, L., et al., *Nucleosome-Chd1 structure and implications for chromatin remodelling.* Nature, 2017. **550**(7677): p. 539-542.
6. Sharma, A., et al., *Crystal structure of the chromodomain helicase DNA-binding protein 1 (Chd1) DNA-binding domain in complex with DNA.* J Biol Chem, 2011. **286**(49): p. 42099-42104.
7. Grune, T., et al., *Crystal structure and functional analysis of a nucleosome recognition module of the remodeling factor ISWI.* Mol Cell, 2003. **12**(2): p. 449-60.
