# Supplementary material for: The role of auxiliary domains in modulating CHD4 activity suggests mechanistic commonality between enzyme families
Source: Nat Commun. 2022 Dec 6;13:7524. doi: 10.1038/s41467-022-35002-0 (PMC9726900; doi:10.1038/s41467-022-35002-0)
Supplement: Supplementary file 3 — Description of additional Supplementary File [file 41467_2022_35002_MOESM3_ESM.pdf]

Descriptions of additional Supplementary Data Files

Supplementary Data 1. CHD4 XLMS data. Crosslinked peptide of CHD4 were analysed by MS. The pLINK output, processed results and final list used for XLMS mapping are included in this excel.
