# Supplementary material for: The role of auxiliary domains in modulating CHD4 activity suggests mechanistic commonality between enzyme families
Source: Nat Commun. 2022 Dec 6;13:7524. doi: 10.1038/s41467-022-35002-0 (PMC9726900; doi:10.1038/s41467-022-35002-0)
Supplement: Supplementary file 5 — Reporting Summary [file 41467_2022_35002_MOESM5_ESM.pdf]

nature portfolio

Corresponding author(s): Joel Mackay

Last updated by author(s): Jun 10, 2022

# Reporting Summary

Nature Portfolio wishes to improve the reproducibility of the work that we publish. This form provides structure for consistency and transparency in reporting. For further information on Nature Portfolio policies, see our Editorial Policies and the Editorial Policy Checklist.

## Statistics

For all statistical analyses, confirm that the following items are present in the figure legend, table legend, main text, or Methods section.

| n/a | Confirmed | |
|---|---|---|
| ☐ | ☒ | The exact sample size (*n*) for each experimental group/condition, given as a discrete number and unit of measurement |
| ☐ | ☒ | A statement on whether measurements were taken from distinct samples or whether the same sample was measured repeatedly |
| ☒ | ☐ | The statistical test(s) used AND whether they are one- or two-sided<br>*Only common tests should be described solely by name; describe more complex techniques in the Methods section.* |
| ☒ | ☐ | A description of all covariates tested |
| ☒ | ☐ | A description of any assumptions or corrections, such as tests of normality and adjustment for multiple comparisons |
| ☒ | ☐ | A full description of the statistical parameters including central tendency (e.g. means) or other basic estimates (e.g. regression coefficient) AND variation (e.g. standard deviation) or associated estimates of uncertainty (e.g. confidence intervals) |
| ☒ | ☐ | For null hypothesis testing, the test statistic (e.g. *F*, *t*, *r*) with confidence intervals, effect sizes, degrees of freedom and *P* value noted<br>*Give P values as exact values whenever suitable.* |
| ☒ | ☐ | For Bayesian analysis, information on the choice of priors and Markov chain Monte Carlo settings |
| ☒ | ☐ | For hierarchical and complex designs, identification of the appropriate level for tests and full reporting of outcomes |
| ☒ | ☐ | Estimates of effect sizes (e.g. Cohen's *d*, Pearson's *r*), indicating how they were calculated |

*Our web collection on statistics for biologists contains articles on many of the points above.*

## Software and code

Policy information about availability of computer code

| | |
|---|---|
| Data collection | Hamamatsu EMCCD camera was used to collect single-molecule movies with their provided software (DCAM-API) and Micromanager 2.0 beta version was used to control camera and shutter during experiments. |
| Data analysis | Single-molecule intensity time trajectories were generated in interactive data language (IDL 8.5) and these trajectories were analyzed in MATLAB using scripts available at https://cplc.illinois.edu/software/<br>Crystallography data was analysed with COOT (version 0.9.6) , PHENIX (1.20. 1-4487-000) and REFMAC (build-in version in CCP4i2 1.0.2).<br>MST data was analysed with MO.Affinity Analysis software v2.3. |

For manuscripts utilizing custom algorithms or software that are central to the research but not yet described in published literature, software must be made available to editors and reviewers. We strongly encourage code deposition in a community repository (e.g. GitHub). See the Nature Portfolio guidelines for submitting code & software for further information.

## Data

Policy information about availability of data

All manuscripts must include a data availability statement. This statement should provide the following information, where applicable:

- Accession codes, unique identifiers, or web links for publicly available datasets
- A description of any restrictions on data availability
- For clinical datasets or third party data, please ensure that the statement adheres to our policy

Data (without normalization) underlying Figure 2C–D, Figure 3A–D, Figure 4A–B, Figure 5C, Figure 6B–C, Figure 7A–B, Supplementary Figure 5, as well as original gel pictures in Figure 2A and Supplementary Figure 7 are provided in the Source Data file.
Database used:
ProteomeXchange: http://www.proteomexchange.org (identifier: PXD033633 )
Crystal structure is in PDB with ID: 8D4Y
Uniprot human reference proteome (UP000005640; May 2020; 20,286 entries)

## Human research participants

Policy information about studies involving human research participants and Sex and Gender in Research.

| | |
|---|---|
| Reporting on sex and gender | N/A |
| Population characteristics | N/A |
| Recruitment | N/A |
| Ethics oversight | N/A |

Note that full information on the approval of the study protocol must also be provided in the manuscript.

# Field-specific reporting

Please select the one below that is the best fit for your research. If you are not sure, read the appropriate sections before making your selection.

☒ Life sciences ☐ Behavioural & social sciences ☐ Ecological, evolutionary & environmental sciences

For a reference copy of the document with all sections, see nature.com/documents/nr-reporting-summary-flat.pdf

# Life sciences study design

All studies must disclose on these points even when the disclosure is negative.

| | |
|---|---|
| Sample size | Sample size was not pre-determined for single molecule assays. Instead, the number of samples required is dictated by the statistical confidence (and experimental design) with which difference between the different FRET states can be identified. In our case, the values of the FRET states can be identified unambiguously by collecting around 70-100 molecules in each conditions tested here. The sample sizes of gel-based assays were chosen based on pilot experiments to ensure that the results would be representative of the true behaviour of the system. Sample size for MST and RTFA assays was chosen based on pilot studies that demonstrated that the reproducibility of the assays was high and that n = 1 was sufficient to provide reliable data. |
| Data exclusions | No data excluded. |
| Replication | 4-6 independent single molecule experiments were carried out to collect enough molecules for our analysis. Each gel-based assay, MST assay, and RTFA assays have been repeated at least 3 times to test reproductivity (all attempt were successful). |
| Randomization | For single molecule assays, molecules were categorized into different FRET states by using a threshold cut off. There is no randomization for gel-based remodelling assays, MST and RTFA assays because there were no relevant experimental groups that required random allocation and no relevant covariates that required controlling. |
| Blinding | In single molecule experiments, blinding is not possible, since molecules are identified using following criteria (1) Photobleaching of dyes or at least acceptor dye must photobleach (2) Anti-correlation change in intensity traces of donor and acceptor and (3) No blinking of dyes must occur. Hence, molecules were analysed manually. For other experiments, blinding is not relevant because no decisions need to be made during data analysis that require experimenter judgment. |

# Reporting for specific materials, systems and methods

We require information from authors about some types of materials, experimental systems and methods used in many studies. Here, indicate whether each material, system or method listed is relevant to your study. If you are not sure if a list item applies to your research, read the appropriate section before selecting a response.

## Materials & experimental systems

| n/a | Involved in the study |
|---|---|
| ☐ | ☒ Antibodies |
| ☐ | ☒ Eukaryotic cell lines |
| ☒ | ☐ Palaeontology and archaeology |
| ☒ | ☐ Animals and other organisms |
| ☒ | ☐ Clinical data |
| ☒ | ☐ Dual use research of concern |

## Methods

| n/a | Involved in the study |
|---|---|
| ☒ | ☐ ChIP-seq |
| ☒ | ☐ Flow cytometry |
| ☒ | ☐ MRI-based neuroimaging |

# Antibodies

| | |
|---|---|
| Antibodies used | Anti-Flag Affinity Gel from Biomake.com; Cat.No. 23102; Clone name: 1E6 Mouse IgG2b; Lot No. 710037. |
| Validation | None |

# Eukaryotic cell lines

Policy information about cell lines and Sex and Gender in Research

| | |
|---|---|
| Cell line source(s) | HEK Expi293F™ cells from Thermo Fisher Scientific, Catalog number: A14527 |
| Authentication | Not tested |
| Mycoplasma contamination | Not tested |
| Commonly misidentified lines (See ICLAC register) | None |
